# Slow magnetic relaxation in a novel carboxylate/oxalate/hydroxyl bridged dysprosium layer†
†Electronic supplementary information (ESI) available. CCDC 1048230 and 1048231. For ESI and crystallographic data in CIF or other electronic format see DOI: 10.1039/c5sc00491h



**DOI:** 10.1039/c5sc00491h

**Published:** 2015-03-17

**Authors:** Dan-Dan Yin, Qi Chen, Yin-Shan Meng, Hao-Ling Sun, Yi-Quan Zhang, Song Gao

**Affiliations:** a Department of Chemistry and Beijing Key Laboratory of Energy Conversion and Storage Materials , Beijing Normal University , Beijing 100875 , P. R. China . Email: haolingsun@bnu.edu.cn; b Jiangsu Key Laboratory for NSLSCS , School of Physical Science and Technology , Nanjing Normal University , Nanjing 210023 , P. R. China . Email: zhangyiquan@njnu.edu.cn; c Beijing National Laboratory for Molecular Sciences , State Key Laboratory of Rare Earth Materials Chemistry and Applications , College of Chemistry and Molecular Engineering , Peking University , Beijing 100871 , China . Email: gaosong@pku.edu.cn

## Abstract

A new 2D dysprosium layer compound has been successfully synthesized from reaction with 2-(3-pyridyl) pyrimidine-4-carboxylic acid (H3-py-4-pmc), in which the Dy^3+^ ions reside in square antiprismatic coordination environments and are connected by carboxylate/oxalate/hydroxyl bridges. Magnetic studies reveal ferromagnetic interactions between Dy^3+^ ions, slow magnetic relaxation with an effective energy barrier *U*_eff_ of 186 K under zero dc field and pronounced hysteresis loops at low temperatures. Further dilution magnetic study suggests that the slow magnetic relaxation originates from the single-ion magnetic behavior of Dy^3+^ ion and that magnetic coupling suppresses the quantum tunneling of magnetization at low temperature. In addition, theoretical calculation indicates strong Ising anisotropy of the Dy^3+^ ion that is due to the strong interaction between Dy^3+^ ions and hydroxyl groups.

## Abstract 2

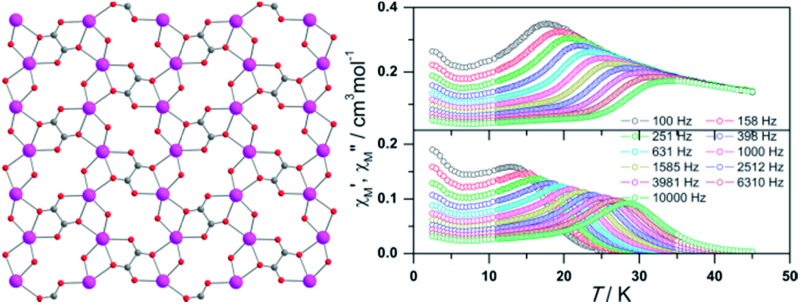
2D dysprosium complex exhibiting slow magnetic relaxation originating from the strong Ising anisotropy of single Dy^3+^ ions has been reported.

## Introduction

Molecular nanomagnets featuring magnetic bistability of molecular origin have gained great recognition due to their fascinating magnetic behaviors and potential applications in functional devices.^1^ For such molecules, most of them display in-phase and out-of-phase ac signals and/or magnetic hysteresis-loops below the blocking temperature. The spin-reversal energy barrier, *U*_eff_, is considered as one of the key factors to evaluate a molecular nanomagnet, which is mainly governed by the magnetic anisotropy of the spin carriers.^2^ It has already been established that the large spin ground state and high spin–orbit coupling of lanthanide ions are apt to bring significant magnetic anisotropy, which can potentially fulfil the conditions to obtain high energy barriers.^3^ Furthermore, the magnetic anisotropy of lanthanide ions is strongly influenced by the ligand field around them, which is proven by the aspherical electron density distribution model and electrostatic model.^4–8^ Therefore, organic ligands play a crucial role in governing the magnetic properties of lanthanide compounds. Up to now, many types of organic ligands, such as phthalocyaninato,^3a,5^ β-diketone,^3d,6^ cyclopentadiene^3c,7^ and Schiff-base ligands^8^ have been successfully utilized for the construction of lanthanide molecular nanomagnets.

According to Pearson's hard–soft acid–base (HSAB) principle, lanthanide ions have a high affinity for oxygen atoms, and carboxylate can generally cater for the oxophilic nature of lanthanide ions.^9^ Additionally, the ability of carboxylate to take different binding modes fits well with the flexible coordination geometries of lanthanide ions, which is proven to be another advantage to build lanthanide–organic frameworks (LnOFs).^10^ Therefore, carboxylate-based ligands have been used widely to construct various LnOFs with aesthetically fascinating architectures and unique luminescent properties.^11^ In contrast to luminescent LnOFs, carboxylate-based LnOFs with interesting magnetic behaviors are relatively rare. Although temperature- and frequency-dependent ac signals have been found in many reported carboxylate-based LnOFs, few of them show clear thermal relaxation processes, which is probably due to the strong quantum tunneling magnetization.^12^ Up until now, the energy barrier of reported carboxylate-based molecular nanomagnets has remained low, which is mainly attributed to the weak coordination field of carboxylate-based ligands.^13^ While the construction of carboxylate-based lanthanide molecular nanomagnets with a high energy barrier remains a challenging task, we hypothesize that the combination of other ligands with strong coordination ability may provide a suitable approach to solving this problem.^14^

Our recent work has focused on the construction of LnOFs by using a novel family of pyrimidine–carboxylate ligands. As a result, a series of lanthanide–pyridine–carboxylate frameworks have been successfully constructed.^15^ In particular, we recently reported a unique 1D carboxylate-bridged chain with slow magnetic relaxation and illustrated the origin of the magnetic relaxation by a dilution method.^13*a*^ As a continuation of the work based on pyrimidine–carboxylates, we present in this paper the synthesis and magnetic study of a novel 2D layer compound based on carboxylate/oxalate/hydroxyl bridges, namely, [Dy(3-py-4-pmc)(C_2_O_4_)_0.5_(OH)(H_2_O)] (**1**), which exhibits slow magnetic relaxation and a pronounced hysteresis loop at low temperatures. The dilution magnetic studies and *ab initio* calculations of **1** indicate that the slow magnetic relaxation originates from the strong Ising anisotropy of single Dy^3+^ ions as a result of the strong interaction between the Dy^3+^ ion and hydroxyl groups.

## Experimental

The ligand 2-(3-pyridyl) pyrimidine-4-carboxylic acid (H3-py-4-pmc) was synthesized according to literature procedures.^16^ Other reagents and solvents employed were commercially available and used as-received without further purification.

### [Dy(3-py-4-pmc)(C_2_O_4_)_0.5_(OH)(H_2_O)] (**1**)

An aqueous solution (5 ml) of H3-py-4-pmc (0.3 mmol) was neutralized with NaOH solution (0.1 mol L^–1^, 3 ml), and solid Dy(NO_3_)_3_·6H_2_O (0.1 mmol) was then added, which was stirred for 10 min at room temperature. The mixture was transferred to a 20 ml Teflon reactor and heated to 135 °C for 72 h under autogenous pressure before cooling to room temperature at a rate of 5 °C h^–1^. Yellowish crystals of **1** were obtained. Yield: 33.0 mg (74.7% based on the metal salt). Elemental analysis (%) calcd for C_11_H_9_DyN_3_O_6_: C, 29.91, H, 2.04, N, 9.52; found C, 29.89, H, 2.18, N, 9.34. IR (KBr, cm^–1^): 3546(s), 1593(s), 1544(s), 1411(m), 1369(s), 754(m), 684(m).

### [Y(3-py-4-pmc)(C_2_O_4_)_0.5_(OH)(H_2_O)] (**2**)

Compound **2** was prepared following a method similar to that of **1**, with the exception of using Y(NO_3_)_3_·6H_2_O instead of Dy(NO_3_)_3_·6H_2_O. Yield: 22.7 mg (61.7%, based on the metal salt). Elemental analysis (%) calcd for C_11_H_9_YN_3_O_6_: C, 35.89, H, 2.44, N, 11.43; found C, 36.21, H, 2.92, N, 11.54. IR (KBr, cm^–1^): 3531(s), 1582(s), 1543(s), 1410(m), 1369(s), 727(m), 684(m).

### [Dy_0.06_Y_0.94_(3-py-4-pmc)(C_2_O_4_)_0.5_(OH)(H_2_O)] (**3**)

An aqueous solution (5 ml) of H3-py-4-pmc (0.3 mmol) was neutralized with a NaOH solution (0.1 mol L^–1^, 3 ml). A 1 ml aqueous solution of Dy(NO_3_)_3_/Y(NO_3_)_3_ (in a 1 : 19 molar ratio; 0.1 mmol in total) was then added to the solution, which was stirred for 10 min at room temperature. The mixture was transferred to a 20 ml Teflon reactor and heated to 135 °C for 72 h under autogenous pressure before cooling to room temperature at a rate of 5 °C h^–1^. Yellowish crystals of **3** were obtained. Yield: 23.8 mg (63.8% based on the metal salt). Elemental analysis (%) calcd for C_11_H_9_Dy_0.06_Y_0.94_N_3_O_6_: C, 35.47, H, 2.44, N, 11.28; found C, 35.77, H, 2.60, N, 11.18.

## Results and discussion

### Crystal structure

**Fig. 1:**
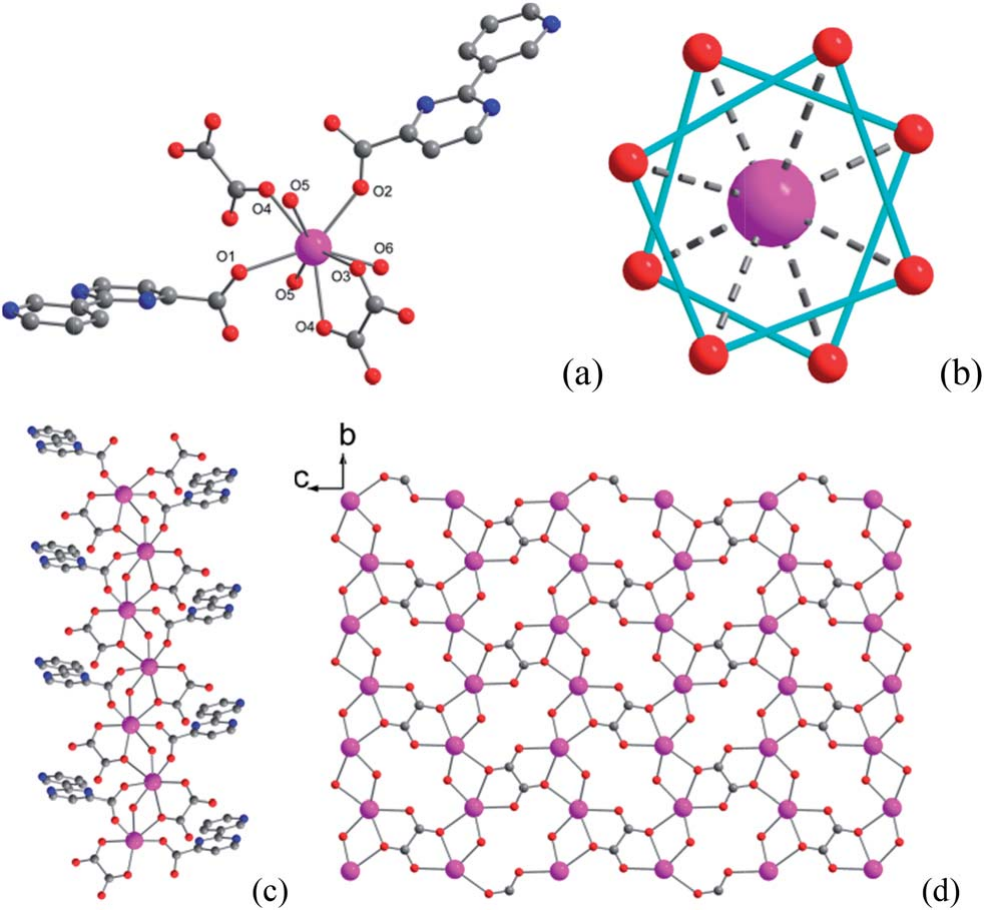
Coordination environment of Dy1 (a), coordination polyhedra around Dy^3+^ (b), 1D chain (c) and 2D layer (d) structure of compound **1**.

Single-crystal X-ray analysis reveals that compound **1** crystallizes in the space group *P*2_1_/*c* and contains a novel two-dimensional (2D) layer structure bridged by hydroxyl, oxalate and 3-py-4-pmc^–^ ligands (Fig. 1, Table S1†). The asymmetric unit of **1** is composed of one Dy^3+^ ion, one 3-py-4-pmc^–^ ligand, half oxalate, one hydroxyl group and one coordination water molecule. As shown in Fig. 1a, each Dy^3+^ ion in **1** is eight-coordinated by two carboxylate oxygen atoms from two 3-py-4-pmc^–^ ligands, three oxalate oxygen atoms from two oxalate, two oxygen atoms from two hydroxyl groups and one water molecule, resulting in an O8 donor set with a distorted square–antiprism (Fig. 1b). The Dy–O distances are between 2.264 and 2.511 Å, similar to those found in reported Dy^3+^ complexes constructed by other carboxylate ligands (Table S2†).^10–14^ The carboxylate groups of 3-py-4-pmc^–^ ligands adopt the *syn*–*syn* bridging mode and connect the adjacent Dy^3+^ ions to produce a 1D chain along the *b* direction with an intrachain Dy···Dy distance of 3.878 Å, which is further furnished by one μ_2_-hydroxyl and one μ_2_:η_3_-(κO, O, κO′)-oxalate bridge (Fig. 1c). It is worth mentioning that the Dy1–O5 bond lengths (2.264 and 2.300 Å) are somewhat shorter than those between Dy1 and other coordination oxygen atoms (2.337–2.511 Å), indicating a stronger interaction between the negative hydroxyl and the positive Dy^3+^ ion, which may play a crucial role in mediating the magnetic behavior of **1**. The adjacent 1D chains are further connected by the oxalate groups in the μ_2_:η_3_-(κO, O, κO′) configuration, resulting in a 2D layer parallel to the *bc* plane with the shortest interchain Dy···Dy distance of 6.283 Å (Fig. 1d). The oxalate is likely derived from the decomposition of H3-py-4-pmc since no oxalate was directly introduced to the reaction, which has also been observed for other oxalate-containing systems.^17^ The adjacent layers are further linked by hydrogen bonds between the coordination water molecules and uncoordinated pyridyl groups of the 3-py-4-pmc^–^ ligand (O6···N3 = 2.727 Å, ∠O6–H6A···N3 = 166.76°), producing a complicated 3D framework (Fig. S1†).

To confirm the phase purity, powder X-ray diffraction (PXRD) analysis of the same samples used for magnetic measurement was carried out at room temperature (Fig. S2†). The diffraction peaks of the as-synthesized samples are in good agreement with the simulated pattern calculated from the single crystal data, demonstrating the high phase purity of the complex.

### Magnetic behavior

**Fig. 2:**
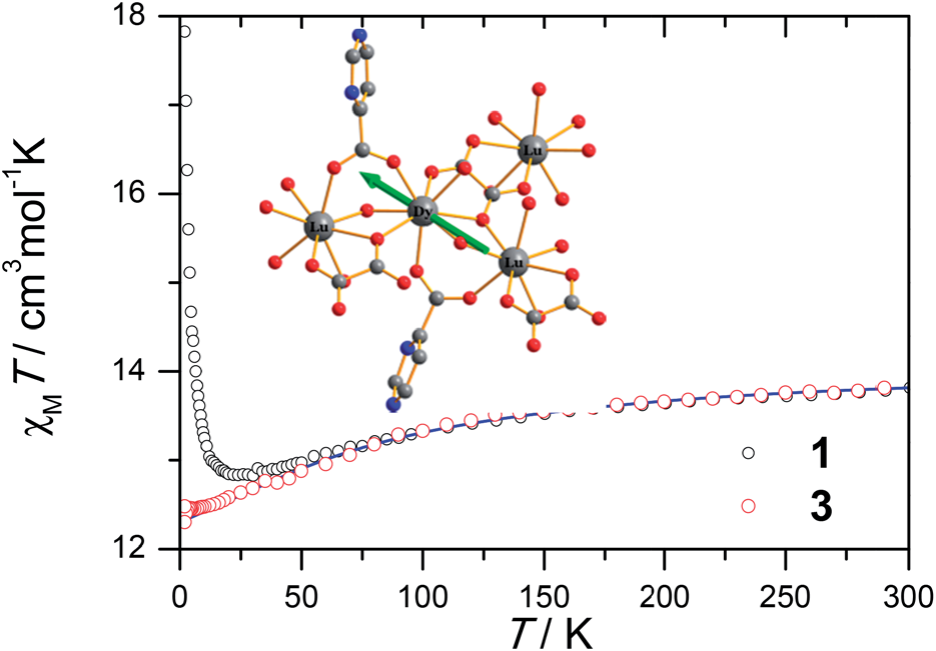
Plots of *χ*_M_ *T* under a 1 kOe dc field for **1** and the diluted sample of **3**. The *χ*_M_ *T* of **3** are divided by the percentage of Dy^3+^ ion. The blue line is the simulation from *ab initio* calculation. Inset: structure of the Dy^3+^ structural fragment; the green arrows represent the magnetic axis calculated by using CASSCF calculation.

The magnetic susceptibility of **1** was measured on microcrystalline samples in the temperature range of 2–300 K at 1 kOe. As shown in Fig. 2, at room temperature, the observed *χ*_M_ *T* value of 13.84 cm^3^ mol^–1^ K is slightly smaller than the expected value (14.2 cm^3^ mol^–1^ K) for one Dy^3+^ ion with a ground state of ^6^ *H*_15/2_.^3–8^ The *χ*_M_ *T* product for **1** gradually goes down in a wide temperature range from 300 to 22 K reaching a minimum of 12.84 cm^3^ mol^–1^ K at 22 K, which is a typical behavior for Dy^3+^ ion due to the progressive depopulation of the excited Stark sublevels.^5–8^ Upon further cooling, the *χ*_M_ *T* value increases sharply to a maximum of 17.82 cm^3^ mol^–1^ K at 2 K, which might be due to the ferromagnetic interaction between Dy^3+^ ions or the occupation of Stark sublevels with higher |*J*z| value at low temperatures. Furthermore, the field dependence of the magnetization was investigated in the range of 0 to 50 kOe at 2, 3, 5, 8 and 10 K, respectively (Fig. S3†). The magnetization at 2 K rapidly increases at low field, indicating well-separated excited Kramer’s doublets. The non-superposition of the *M vs. H*/*T* plots at higher fields implies the presence of significant magnetic anisotropy in **1**.

**Fig. 3:**
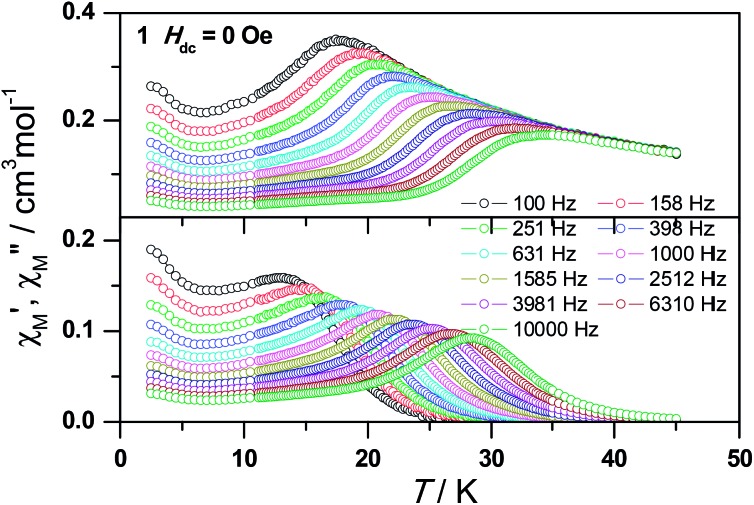
Frequency dependence of the in-phase (top) and out-of-phase (bottom) ac susceptibility signals under zero field for **1**.

**Fig. 4:**
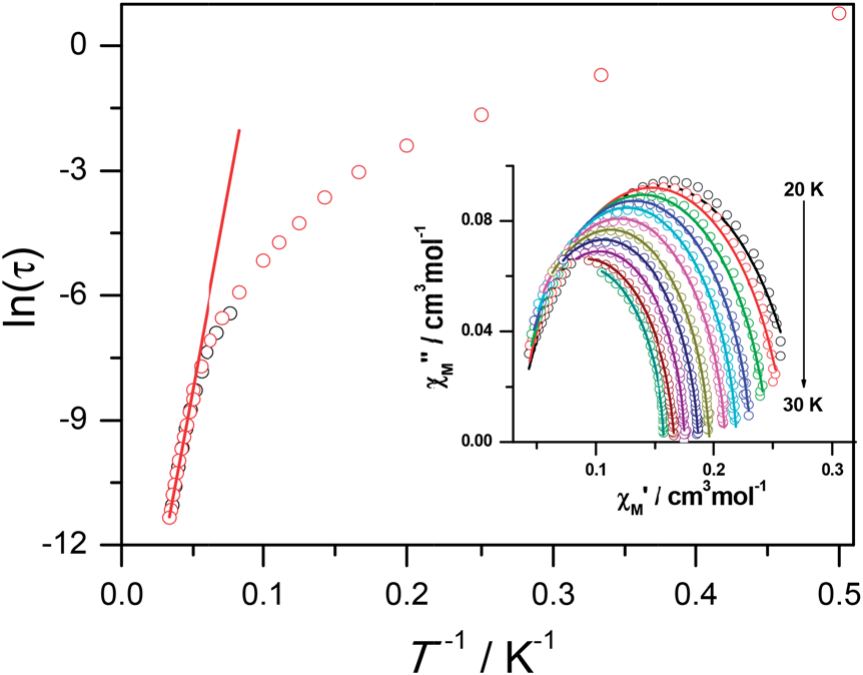
Plot of ln *τ vs. T* ^–1^ for **1**. The black and red circles represent the relaxation time extracted from temperature and frequency dependence *ac* data respectively. The red lines show the fitting results according to the Arrhenius law. Inset: the fitting of ac-f data by a generalized Debye model.

Ac susceptibility measurements were conducted under a zero dc field for **1** to further explore the dynamics of magnetization. As shown in Fig. 3, both the in-phase (*χ*′M) and out-of phase (*χ*′′M) signals of ac susceptibilities are strongly frequency dependent below 40 K, indicating the slow relaxation of magnetization. Both the in-phase and out-of phase susceptibilities clearly show frequency-dependent peaks, indicating the “freezing” of the spins by the anisotropy barriers. With increasing ac frequency, the peaks gradually move to the high temperature region. It is noteworthy that the *χ*′′M plots at 10 kHz show a maximum at 28.62 K, which is fairly high as compared to the majority of lanthanide molecular nanomagnets. Upon further cooling, the upturns of *χ*′M and *χ*′′M are observed below 7 K, implying that the quantum tunneling mechanism plays a dominant role. The relaxation time can be extracted from both the temperature- and frequency-dependent ac data at different temperatures (Fig. S4, Table S3 and S4†). In the high temperature region, the relaxation process of **1** follows an Arrhenius law with an effective barrier *U*_eff_ = 186 K and with a pre-exponential factor of 2.44 × 10^–8^ s. In addition, the fitting of ac-f data by a generalized Debye model gives rise to *α* parameters of 0.071–0.14, indicating a single relaxation process mainly involved at 20–30 K, which is consistent with the presence of a unique coordination sphere of Dy^3+^ in **1** (Fig. 4).

The quantum tunnelling of magnetization (QTM) at low temperature mainly originates from the hyperfine interaction of the Dy^3+^ ion with the nuclear spin at low temperature so that the Kramer’s ion nature of the Dy^3+^ breaks down and the total quantum number in the ground state is split by the low symmetry.^5*d*^ To suppress the quantum tunneling process below 7 K, ac measurements under 2 kOe optimum dc field were carried out at various frequencies (Fig. S5a†). As shown in Fig. S5b,† the upturn of *χ*′′M almost disappears, indicating that quantum tunneling is dramatically reduced. Meanwhile, the peak temperature of *χ*′′M slightly shifts from 28.62 K to 28.82 K at 10 kHz. The data obtained at high temperatures nicely follow a thermally activated behavior with an effective energy barrier of *U*_eff_ = 210 K and *τ*_0_ = 1.25 × 10^–8^ s based on the Arrhenius law and the former is slightly higher than that under zero dc field (Fig. S6†). The *U*_eff_ values for **1** obtained at zero and applied dc fields are significantly higher than those reported for any carboxylate-based 4f SMMs, which is ascribed mainly to the strong Ising-axis magnetic anisotropy of Dy^3+^ centers arising from the strong coordination ability of hydroxyl groups.

**Fig. 5:**
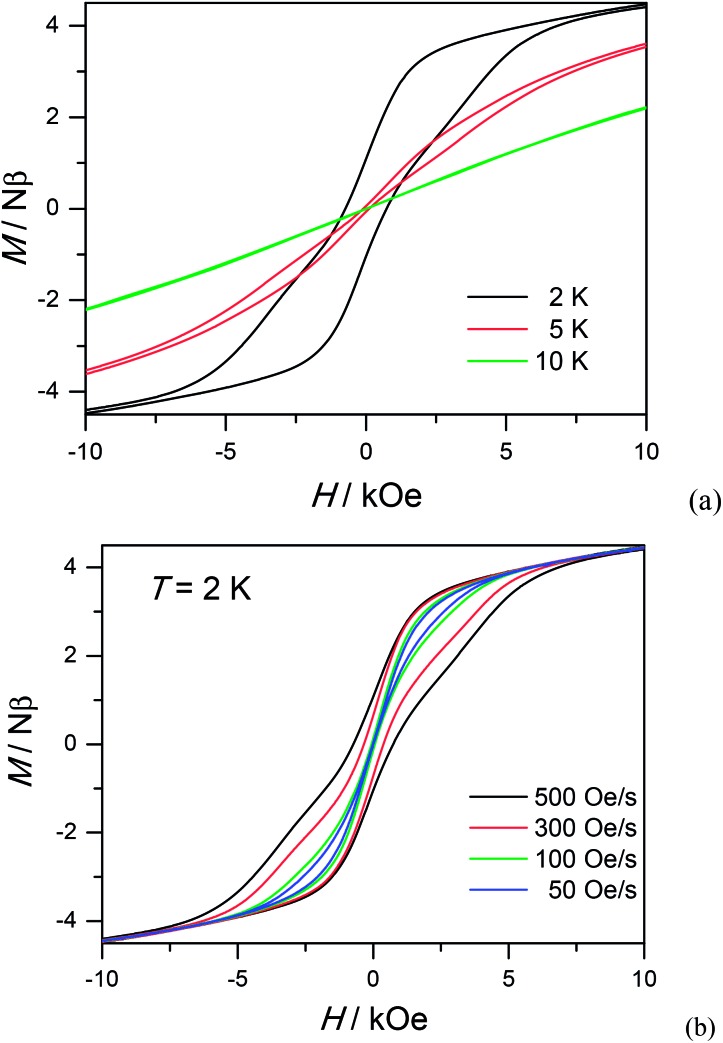
Hysteresis loop for **1** measured at different temperatures with sweep rates of 500 Oe s^–1^ (a) and different sweeping rates at 2 K (b).

To study further the dynamic behavior of complex **1**, low-temperature hysteresis loops were measured on microcrystalline samples using a SQUID-VSM magnetometer. As shown in Fig. 5a, butterfly-shaped loops are clearly visible below 5 K within ± 10 kOe at a sweeping rate of 500 Oe s^–1^, which is typical for lanthanide molecular nanomagnets. When the temperature goes down to 2 K, the butterfly-shaped hysteresis loop becomes much larger and wider with a coercive field (*H*_c_) of 720 Oe at 2 K. The loop at 2 K does not show a clear fast-tunnel step around zero dc field, indicating that QTM is not dominant in this situation. As the field increases, the loops become broader with a maximum opening occurring at *ca.* 2 kOe, which is fairly close to the ac optimum field of 2 kOe. Upon decreasing the sweeping rate, QTM becomes more obvious and the loops become narrower, also indicating the dynamic behavior of **1** (Fig. 5b). The strong dependence of hysteresis loops on the temperature and field sweep rates is in good agreement with that observed in the ac susceptibility data.

### Dilution magnetic studies

To obtain further evidence on the origination of slow magnetic relaxation, we synthesized the diamagnetic yttrium analogue [Y(3-py-4-pmc)(C_2_O_4_)_0.5_(OH)(H_2_O)] (**2**) and doped the diamagnetic lattice with paramagnetic Dy^3+^ ions to get a diluted sample [Dy_0.06_Y_0.94_(3-py-4-pmc)(C_2_O_4_)_0.5_(OH)(H_2_O)] (**3**). The exact amount of Dy^3+^ ion was confirmed by inductively coupled plasma (ICP) measurements that allow trace concentrations of an element in a sample to be precisely determined. This reveals a 6% dysprosium dilution, which is slightly higher than the value of 5% employed in the synthesis. X-Ray single-crystal and powder diffraction data indicate that compounds **2** and **3** are iso-structural with **1** (Table S1, Fig. S2†). Furthermore, the X-ray powder diffraction pattern of **3** matches nicely with that found for **1** as well as the simulation pattern from single crystal data of **1**, thus confirming the phase purity of the bulky samples used for the magnetic measurements.

**Fig. 6:**
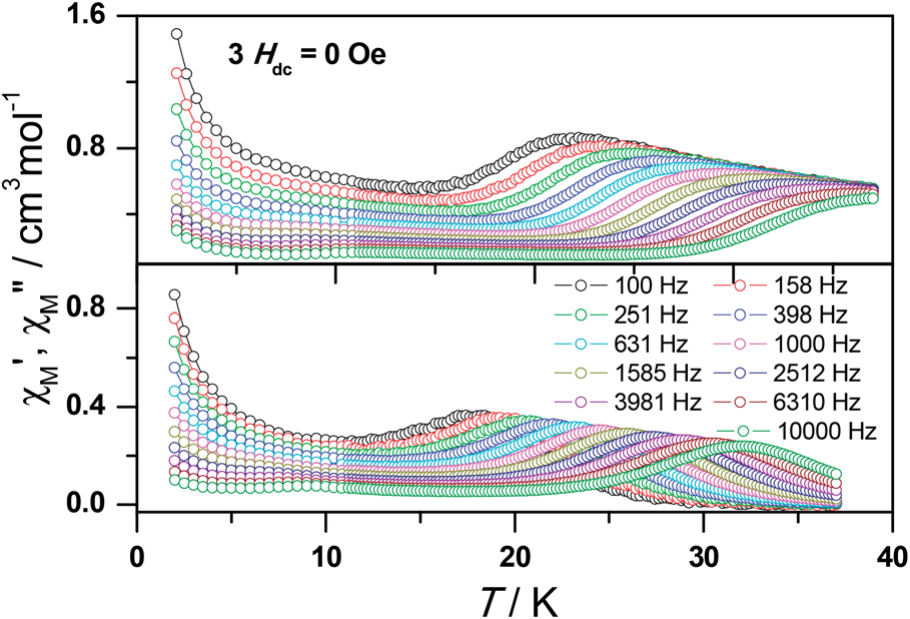
Frequency dependence of the in-phase (top) and out-of-phase (bottom) ac susceptibility signal under zero field for a diluted sample of **3**.

Temperature-dependent dc susceptibilities of **3** were also measured (Fig. 2). It can be seen clearly that the *χ*_M_ *T* value descends very slowly upon cooling, which is a typical behavior of 4f paramagnetic ions.^5–8^ The very small declines of the *χ*_M_ *T* products suggest that the excited Kramer’s doublets are very well isolated from the ground state. Below 12 K, a rapid fall of *χ*_M_ *T* is observed, which is quite different from the increase of the *χ*_M_ *T* value below 22 K found for **1**, indicating the existence of magnetic blocking and the weakening of magnetic interactions by virtue of dilution. Ac susceptibility measurements were also performed. As shown in Fig. 6, the diluted compound **3** exhibits a strong temperature- and frequency-dependent behavior under zero dc field, suggesting the single-ion origin of such a slow relaxation. Similar to **1**, compound **3** also shows two relaxation processes, one of which is the thermally-activated one and the other is quantum tunneling. Compared with **1**, the tail of *χ*′′M for **3** below 10 K from quantum tunneling increases greatly at nearly all frequencies, indicating that the magnetic coupling between Dy^3+^ ions in **1** can suppress the quantum tunneling effect, which is quite different to that found in our ferromagnetic 1D system.^15*b*^ The relaxation time of **3** can also be extracted from both the temperature- and frequency-dependent ac data at different temperatures (Fig. 6 and Table S5 and S6†). Below 8 K, the dynamics of **3** become almost temperature-independent as expected in a pure quantum regime and the relaxation time falls in the range of 0.011 to 0.0144 s, which are shorter than that found for **1** (0.014–2.18 s), further conforming the suppression of QTM by magnetic coupling (Table S3 and S5†). Above 18 K, the relaxation time can be well fitted by the Arrhenius law, giving rise to *U*_eff_ = 215 K and *τ*_0_ = 2.54 × 10^–8^ s (Fig. S7 and S8†) that are comparable to those found for the undiluted samples of **1**, further confirming the intrinsic single ion magnetic behavior of complex **1**. Similar to **1**, under an optimum dc field of 2 kOe (Fig. S9a†), the low temperature quantum tunneling effect can also be suppressed and the high temperature data nicely follows the Arrhenius law with an effective energy barrier of *U*_eff_ = 245 K and *τ*_0_ = 8.28 × 10^–9^ s (Fig. S9 and S10†). The hysteresis loops of **3** can also be observed at low temperatures (Fig. S11†). At 2 K, the loop shows a clear and fast tunnel step around zero dc field, indicating that quantum tunneling becomes dominant at this temperature, which is consistent with that observed in the ac susceptibility data under zero dc field. Similar to **1**, the shape of the loops at 2 K are greatly affected by the field sweep rate, further supporting the dynamic behavior of **3**. Taken together, the in-depth study of the diluted sample clearly demonstrates that the slow relaxation of the magnetization originates from the single-ion magnetic behavior of Dy^3+^ ions and the magnetic interaction between them can suppress the QTM at low temperatures.

### Electronic structure calculations

**Fig. 7:**
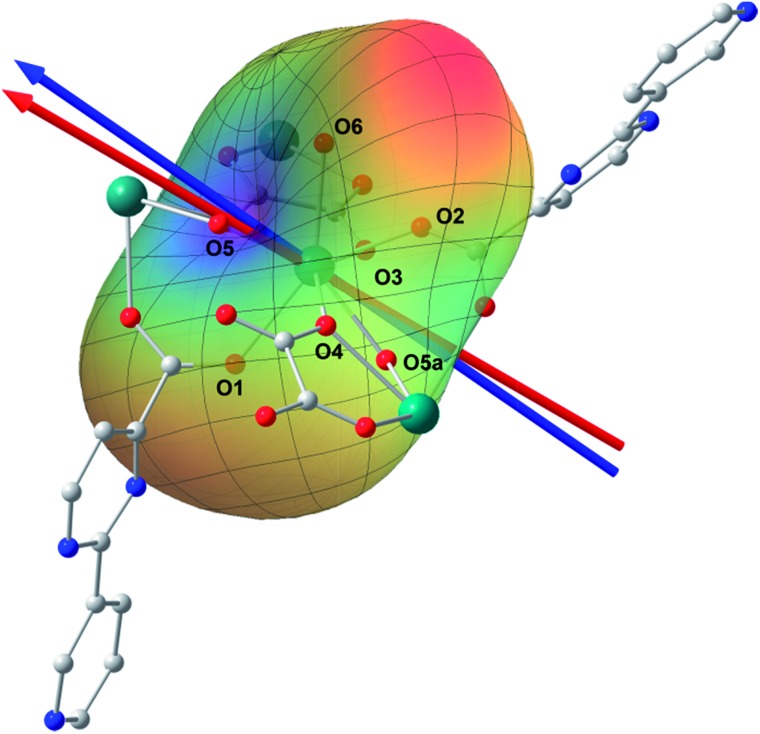
Structure of a Dy^3+^ structural fragment together with the charges of the first coordination sphere; the red and blue arrows represent the magnetic axis calculated by using CASSCF and an electrostatic model (see the text) calculation, respectively. The rainbow surface describes the potential energy of the Ising limit state of ^6^ *H*_15/2_ in the electrostatic field generated by all the atoms, where blue indicates a low potential and red represents a high potential. H atoms have been omitted for clarity.

*Ab initio* calculation has recently proven to be an efficient and powerful method in magnetic anisotropic investigation of rare earth ions.^4e,7c,8a,18^ CASSCF/RASSI/SINGLE_ANSIO calculations on individual dysprosium fragments extracted from complex **1** on the basis of X-ray determined geometries, without symmetry and magnetic interaction considerations, were carried out using the MOLCAS 7.8 and SINGLE_ANISO program package (inset of Fig. 2 and Table S5†).^19,20^ The results provide the effective *g* tensor of the ground Kramer’s doublets on the dysprosium sites as strongly axial (*g*_*x*_ = 0.0019, *g*_*y*_ = 0.0024, *g*_*z*_ = 19.83), which is very close to its Ising limit state. The magnetic easy axis is calculated to be along the direction of Dy–O5, as plotted in the red axis in Fig. 7. The magnetic susceptibility was calculated according to the lowest Russell–Saunders multiplet splitting based on the CASSCF results. The *χ*_M_ *T* value is reasonably consistent with the experimental data on powder samples of **1** above 50 K and nearly all temperature regions for **3**, indicating the validity of the *ab initio* result from the single ion anisotropy point of view. Nevertheless, the inconformity at lower temperatures is probably due to the magnetic interaction between adjacent dysprosium ions.

The CASSCF calculation provides the charge of each atoms around the central Dy^3+^ ion, affording the possibility of understanding the magnetization easy axis orientation. The quantized axis of a rare earth ion located in a crystal field can be predicted by minimizing the interaction potential of the anisotropic electron cloud of a lanthanide ion and its environment. This method was proposed by Chilton and co-workers.^4*b*^ The description of the anisotropic electron cloud employs the spherical harmonics expansion up to the 6th rank proposed by Sievers.^21^ The potentials were calculated according to Hutchings' publication with the charges on all the atoms in the *ab initio* calculation fragment as shown in Fig. 7.^22^ For clarity, only the first coordination sphere charges are marked.

It has been shown that the calculated magnetic easy axis is very close to the direction of Dy–O5 with a bond length of 2.264 Å, the shortest one in the first coordinating sphere. Moreover, this oxygen atom from a hydroxyl group possesses the highest negative charge among all the atoms from the fragment taken into calculation (Table S6†). One can therefore understand the aforementioned orientation of the magnetic easy axis. The shorter length and larger negative charge is able to generate more intense electrostatic potential interaction with the negatively charged electron cloud of the dysprosium ion. The oblate Ising limit electron cloud accordingly prefers to orient its quantized axis along this strong negative charge so as to reduce the interaction between the fat equator plane and the negative charge. The overall effect of the charges minimizes the potential energy when the quantized axis of Dy^3+^ points along the direction of Dy–O5 (the area in blue on the potential surface), while it maximizes the interaction when the quantized axis points to the neutral O6 (the red area). It is necessary to stress that the final orientation of the principal axis is an overall result of all the charges and covalent bond effects, while the above analysis provides a very brief understanding of the electrostatic picture.

To get more insight on the magnetic interactions between the Dy^3+^ ions, program POLY_ANISO^20^ was used to fit the experimental magnetic susceptibilities for obtaining the magnetic coupling parameters *J*_1_, *J*_2_ and *J*_3_ (see eqn S1 and Fig. S12 in the ESI†) within the Lines model.^23^ The total coupling parameters *J*_1_, *J*_2_ and *J*_3_ (dipolar and exchange) were included to fit the magnetic susceptibilities. The calculated and experimental magnetic susceptibilities of complex **1** are shown in Fig. S13,† respectively, where a slight discrepancy of the fit to the experimental data for **1** is found in the low temperature regime for the reason that the long relaxation times make the measured *χ*_M_ *T* lower than the isothermal susceptibility.^24^ The obtained *J*_1_, *J*_2_ and *J*_3_ (*J* = *J* ^dipolar^ + *J* ^exch^) are shown in Table S7 and S8.† Compared with *J*_2_ and *J*_3_, the dipolar part of *J*_1_ is 4.2 cm^–1^, which is the largest due to the shortest Dy···Dy distances (3.877 Å) within the layers, and thus dominates the overall magnetic behavior.

The theoretical calculation of the local anisotropy of Dy^3+^ ions and the magnetic coupling between them provide us a good opportunity to get more insight about the magnetic structure of this 2D layer compound. Within the 2D layer parallel to *bc* plane, the easy axes of Dy^3+^ ions are arranged in a parallel ABAB fashion, resulting in an overall ferromagnetic coupling that causes the increase of *χ*_M_ *T* at low temperatures (Fig. S14†). It is interesting to note that all the easy axes of Dy^3+^ ions reside in the 2D layer bridged by carboxylate, oxalate, and hydroxyl groups, resulting in an interesting 2D Ising model that has rarely been reported before.

## Conclusion

In summary, a novel 2D dysprosium layer compound constructed from pyrimidine-carboxylate, oxalate and hydroxyl ligands has been synthesized and fully characterized. This complex clearly shows slow magnetic relaxation at zero dc field with an energy barrier of 186 K and a pronounced hysteresis loop at low temperatures. The magnetic behavior of **1** is thoroughly studied by a magnetic dilution method and theoretical calculations, which indicate that the thermally activated slow relaxation of the magnetization originates from the single ion magnetic behavior of Dy^3+^ itself. Furthermore, to the best of our knowledge, the energy barrier of complex **1** is the largest among carboxylate containing complexes, which might be due to the quite short distances between Dy^3+^ ions and hydroxyl oxygen atoms.
