# Supplementary material for: Slow magnetic relaxation in a novel carboxylate/oxalate/hydroxyl bridged dysprosium layer
Source: Chem Sci. 2015 Mar 17;6(5):3095–101. doi: 10.1039/c5sc00491h (PMC5490050; doi:10.1039/c5sc00491h)
Supplement: Supplementary file 1 [file SC-006-C5SC00491H-s001.pdf]

Electronic Supplementary Material (ESI) for Chemical Science.
This journal is © The Royal Society of Chemistry 2015

Supporting Information for

Slow magnetic relaxation in a novel carboxylate/oxalate/hydroxyl bridged dysprosium layer

Dan-Dan Yin, ‡ [a] Qi Chen, ‡ [a] Yin-Shan Meng, [c] Hao-Ling Sun [a]*,Yi-Quan Zhang, [b]* and Song Gao[c]*

[a] *Department of Chemistry and Beijing Key Laboratory of Energy Conversion and Storage Materials, Beijing Normal University, Beijing 100875, P. R. China. E-mail: haolingsun@bnu.edu.cn;*

[b] *Jiangsu Key Laboratory for NSLSCS, School of Physical Science and Technology, Nanjing Normal University, Nanjing 210023, P. R. China. E-mail: zhangyiquan@njnu.edu.cn;*

[c] *Beijing National Laboratory for Molecular Sciences, State Key Laboratory of Rare Earth Materials Chemistry and Applications, College of Chemistry and Molecular Engineering, Peking University, Beijing 100871, China. E-mail: gaosong@pku.edu.cn;*

Experimental Section

X-ray crystallography and physical measurement

Intensity data for crystal of **1** was collected on a Bruker Smart Apex Ⅱ CCD diffractometer with graphite-monochromated Mo Kα radiation(0.71073 Å) at 296K. The structures were solved by direct methods and refined with the full-matrix least-squares technique based on F^2 using the SHELXL program. All non-hydrogen atoms were refined anisotropically. Hydrogen atoms were placed at the calculation positions. The details of crystallographic data and selected bond parameters for compounds **1** are listed in Table S1 and Table S2, respectively.

Elemental analyses of carbon, hydrogen, and nitrogen were carried out with an Elementar Vario EL analyzer. FTIR spectra were recorded in the range of 4000 to 400cm^{-1} on an AVATAR 360 Nicolet 380 FT/IR spectrometer using KBr pellets. Powder X-ray diffraction (XRD) analyses were performed on a Rigaku Dmax-2000 X-ray diffractometer with Cu Kα (λ=1.54059 Å) radiation. Variable-temperature magnetic susceptibility measurements of **1** were performed on SQUID-XL, SQUID-VSM and PPMS magnetometer.

Table S1. Crystallographic Data and Structure Refinement for complex **1**and **2**

	**1**	**2**
Formula	$C_{11}H_9DyN_3O_6$	$C_{11}H_9YN_3O_6$
Mr	441.71	368.11
Crystal system	Monoclinic	Monoclinic
Space group	$P\,2_1/c$	$P\,2_1/c$
a(Å)	13.672(1)	13.655(1)
b(Å)	7.313(1)	7.278(1)
c(Å)	12.481(1)	12.428(1)
a(°)	90	90
β (°)	94.296(1)	94.424(1)
γ(°)	90	90
V (Å^3)	1244.4(2)	1231.4(2)
Z	4	4
μ (mm^{-1})	6.039	4.770
F(000)	840	732
GOF	1.065	1.082
Data collected	6510	6473
Unique	2428	2046
R_{int}	0.0186	0.0208
$R1, wR2[I > 2\sigma(I)]$	0.0174, 0.0471	0.0248, 0.0735
R1, wR2 [all data]	0.0186, 0.0480	0.0300, 0.0763

Table S2. Selected Bond Distances (Å) in complex **1**

Dy1-O1	2.445(2)	Dy1-O2c	2.337(2)	Dy1-O3	2.404(2)
Dy1-O4a	2.511(2)	Dy1-O4d	2.482(2)	Dy1-O5	2.300(2)
Dy1-O5b	2.264(2)	Dy1-O6	2.375(2)		

Table S3 Relaxation fitting parameters from Least-Squares Fitting of $\chi(f)$ between 1-1000Hz data under zero dc field of **1**.

Temperature	χ_T	χ_s	α	τ
2K	17.79	0.092	0.50	2.18
3 K	8.47	0.069	0.52	0.49
4 K	5.22	0.066	0.51	0.19
5 K	3.70	0.068	0.49	0.091
6 K	2.81	0.075	0.47	0.048
7 K	2.23	0.081	0.44	0.026
8 K	1.83	0.096	0.40	0.014
9 K	1.55	0.11	0.35	8.82E-3
10 K	1.35	0.12	0.30	5.69E-3
12 K	1.07	0.14	0.22	2.65E-3
14 K	0.90	0.15	0.16	1.42E-3
16 K	0.78	0.17	0.086	8.38E-4
18 K	0.69	0.16	0.061	4.52E-4
20 K	0.62	0.16	0.021	2.55E-4

Table S4 Relaxation fitting parameters from Least-Squares Fitting of $\chi(f)$ between 100-10000 Hz data under zero dc field of **1**.

Temperature	χ_T	χ_S	α	τ
20K	0.28	0.033	0.17	2.04E-4
21K	0.26	0.033	0.13	1.5E-4
22K	0.25	0.033	0.11	1.10E-4
23K	0.23	0.033	0.082	8.2E-5
24K	0.22	0.033	0.061	6.19E-5
25K	0.21	0.030	0.069	4.64E-5
26K	0.20	0.028	0.060	3.45E-5
27K	0.19	0.026	0.063	2.61E-5
28K	0.18	0.030	0.033	2.05E-5
29K	0.17	0.017	0.078	1.42E-5
30K	0.16	0.021	0.049	1.18E-5

Table S5. Relaxation fitting parameters from Least-Squares Fitting of $\chi(f)$ data between 1-1000 Hz under zero dc field of **3**.

T/K	χ_T	χ_S	α	τ
2K	4.64	0.086	0.53	0.0144
3 K	3.31	0.035	0.55	0.0152
4 K	2.59	0.025	0.57	0.0141
5 K	2.18	0.027	0.58	0.0133
6 K	1.90	0.027	0.59	0.0117
7 K	1.73	0.034	0.60	0.0108
8 K	1.63	0.050	0.60	0.0110
14 K	1.12	0.33	0.14	0.00689
16 K	0.97	0.27	0.10	0.00334
18 K	0.86	0.21	0.097	0.00158
20 K	0.78	0.16	0.093	7.76E-4
22 K	0.71	0.14	0.072	3.98E-4
24 K	0.65	0.10	0.087	1.98E-4

Table S6. Relaxation fitting parameters from Least-Squares Fitting of $\chi(f)$ data between 100-10000 Hz under zero dc field of **3**.

T/K	χ_T	χ_S	α	τ
18	1.73	0.12	0.42	0.00438
19	1.40	0.13	0.34	0.00196
20	1.19	0.13	0.28	0.00104
21	1.05	0.13	0.22	6.19E-4
22	0.99	0.13	0.17	4.16E-4
23	0.90	0.13	0.14	2.80E-4
24	0.84	0.13	0.11	2.0E-4
25	0.80	0.13	0.094	1.44E-4
26	0.77	0.12	0.087	1.05E-4
27	0.73	0.12	0.071	7.78E-5
28	0.70	0.12	0.067	5.78E-5
29	0.68	0.12	0.047	4.33E-5
30	0.66	0.11	0.071	3.19E-5
31	0.63	0.11	0.06	2.47E-5
32	0.61	0.10	0.064	1.83E-5

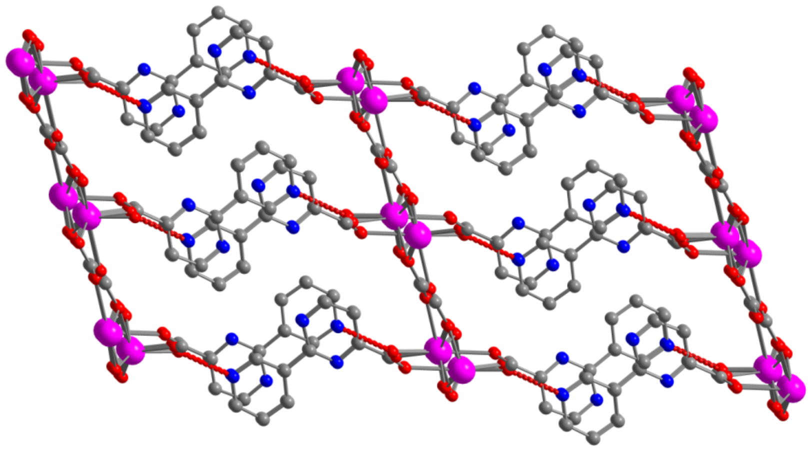

Fig S1. 3D supramolecular structure of compound **1** constructed by hydrogen bonds.

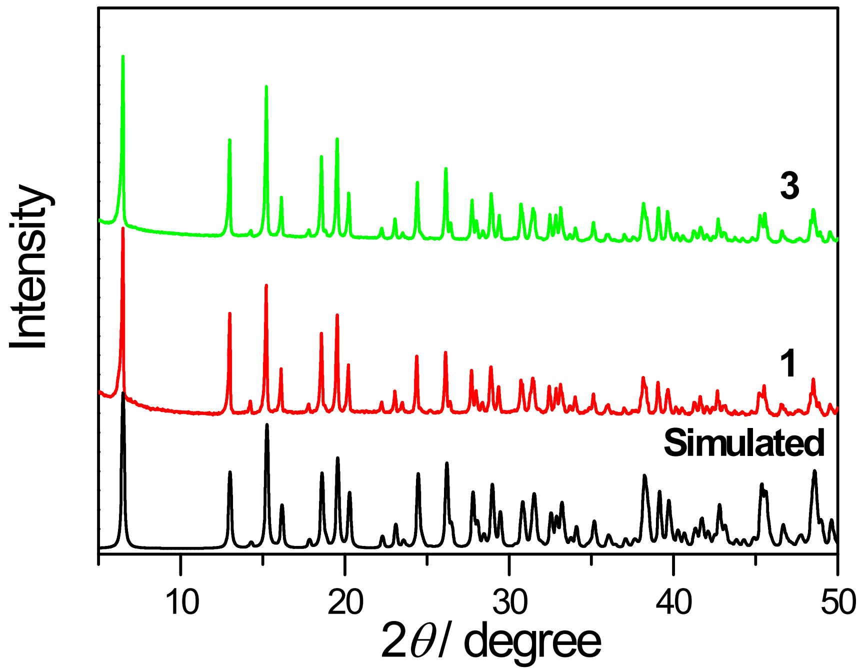

Fig. S2 Powder X-ray diffraction profiles of **1** and **3** together with a simulation from the single crystal data.

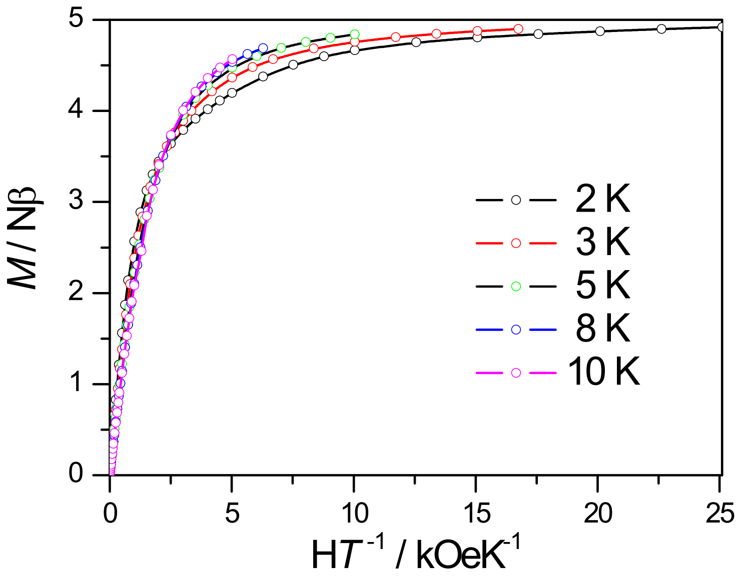

Fig. S3 Plots of *M-H* for **1** at 2, 3, 5, 8 and 10K, respectively.

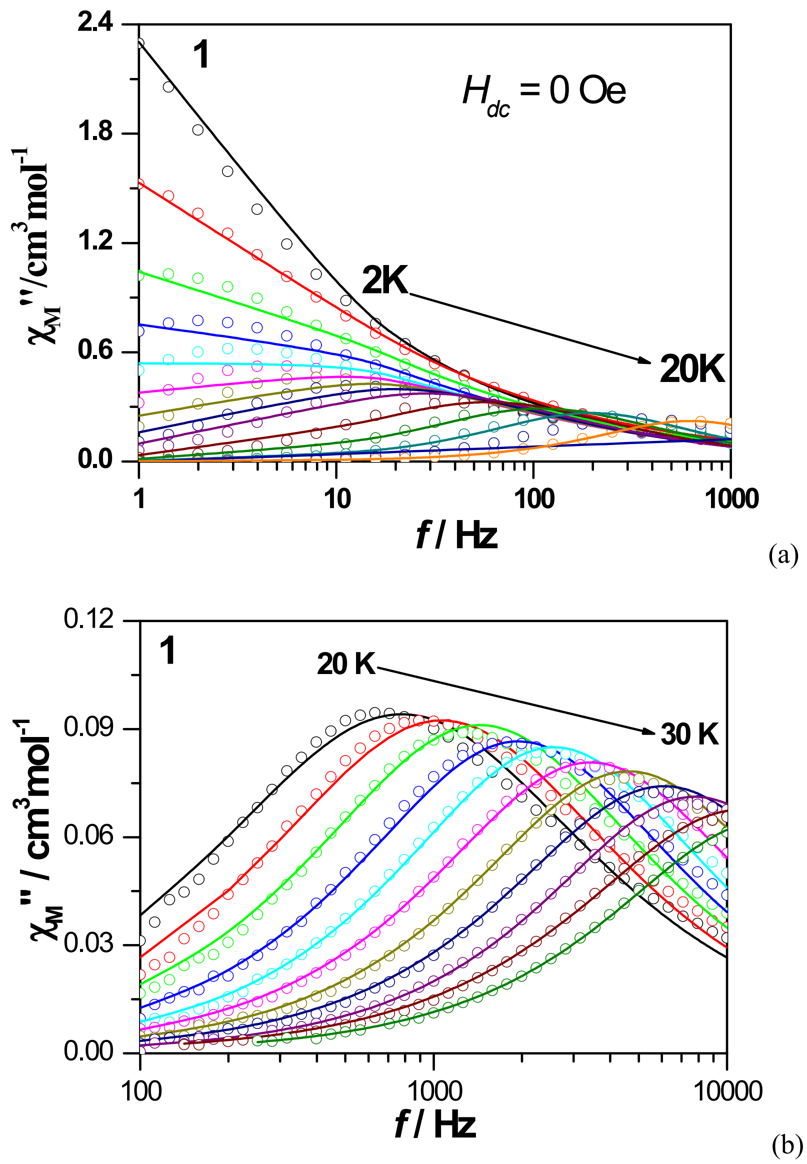

Fig. S4 χ'' (*f*) curves measured under zero *dc* fields for **1** at selected temperatures. Solid lines were fitted using a generalized Debye relaxation model

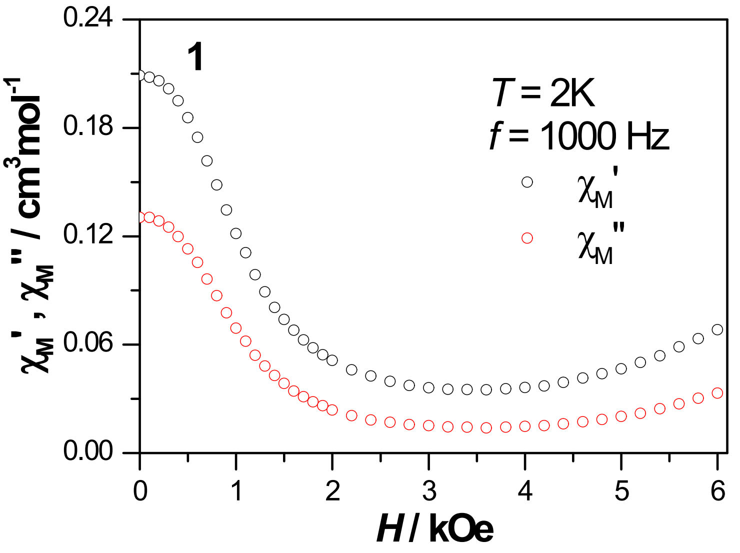

(a)

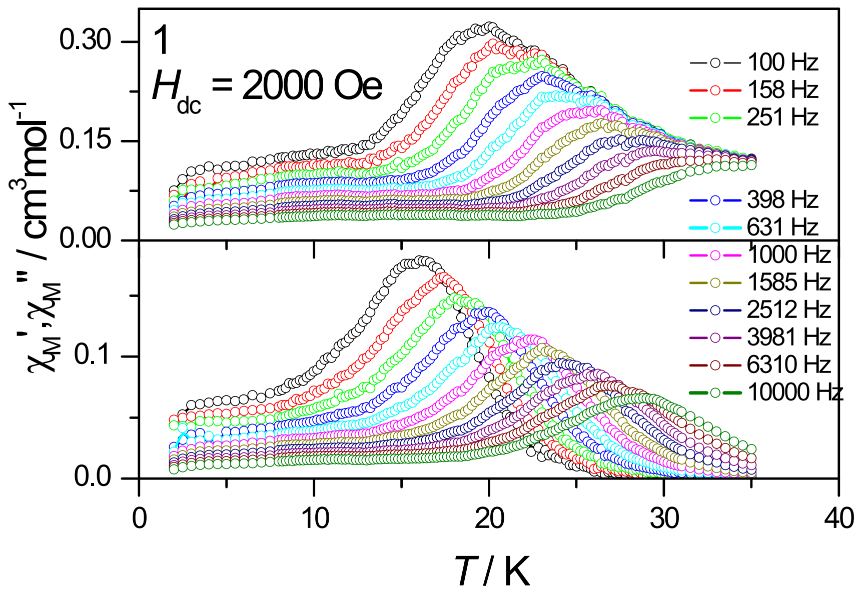

(b)

Fig. S5 The field dependence and temperature dependence of ac susceptibility at 2K for 1 kHz (a) and under 2 kOe field (b) for **1.**

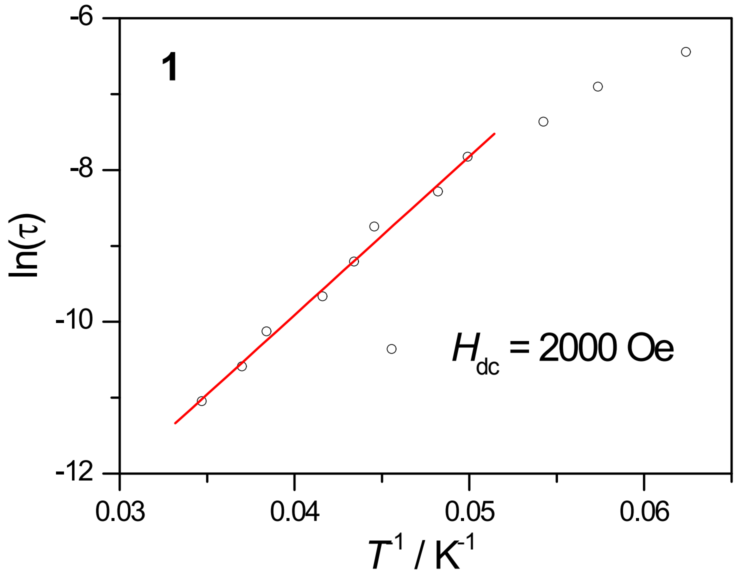

Fig. S6 Plots of lnτ vs. T^{-1} for **1** under 2 kOe dc field. The red lines show the fitting results according to the Arrhenius law.

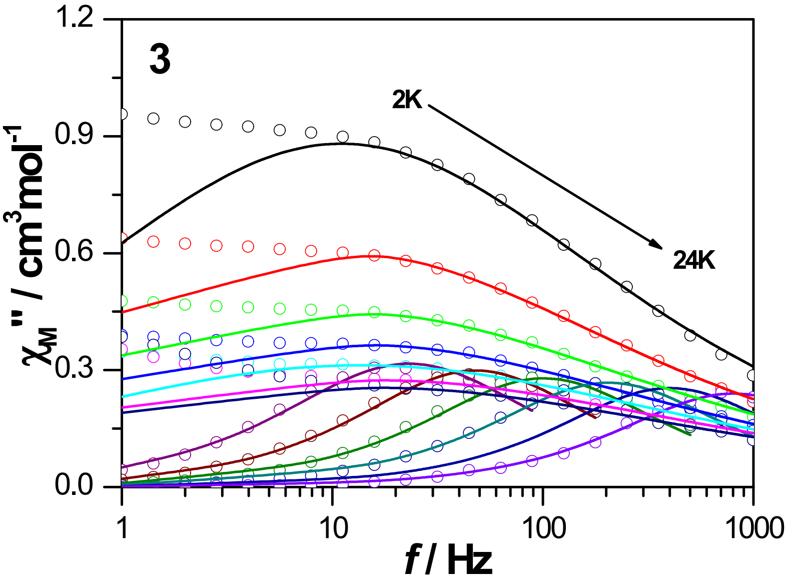

(a)

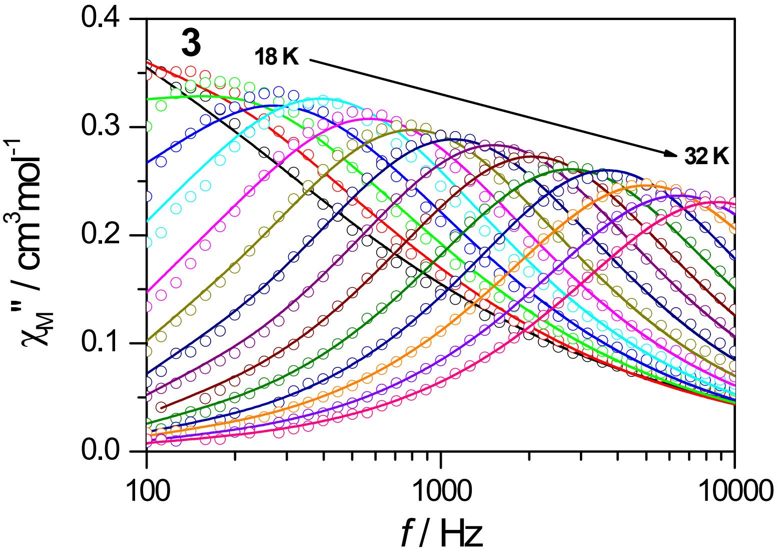

(b)

Fig. S7 χ" (*f*) curves measured under zero *dc* fields for **3** at selected temperatures. Solid lines were fitted using a generalized Debye relaxation model

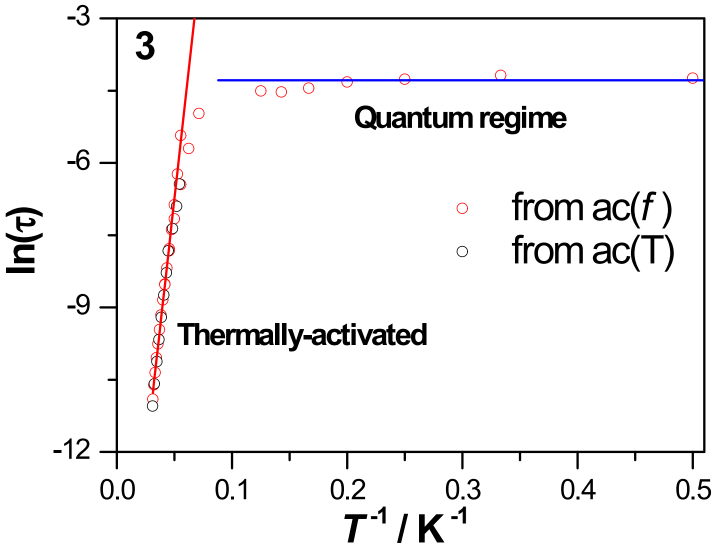

Fig. S8 Plots of lnτ verus T^{-1}. The red lines show the fitting results according to the Arrhenius law.

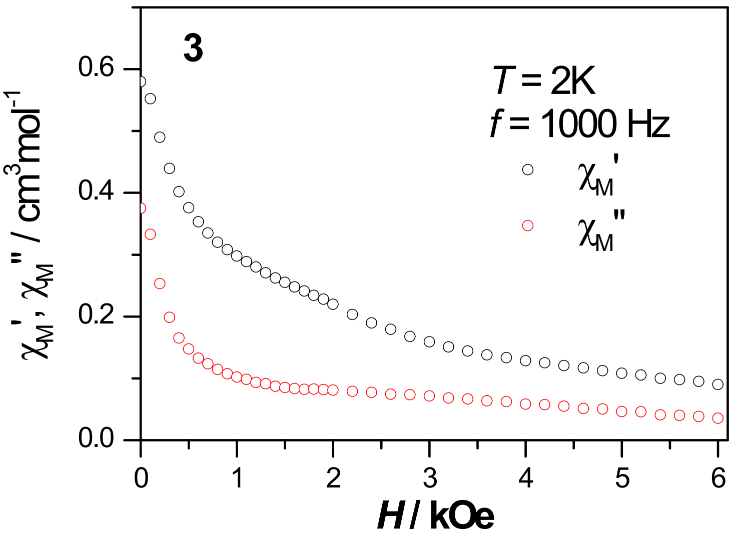

(a)

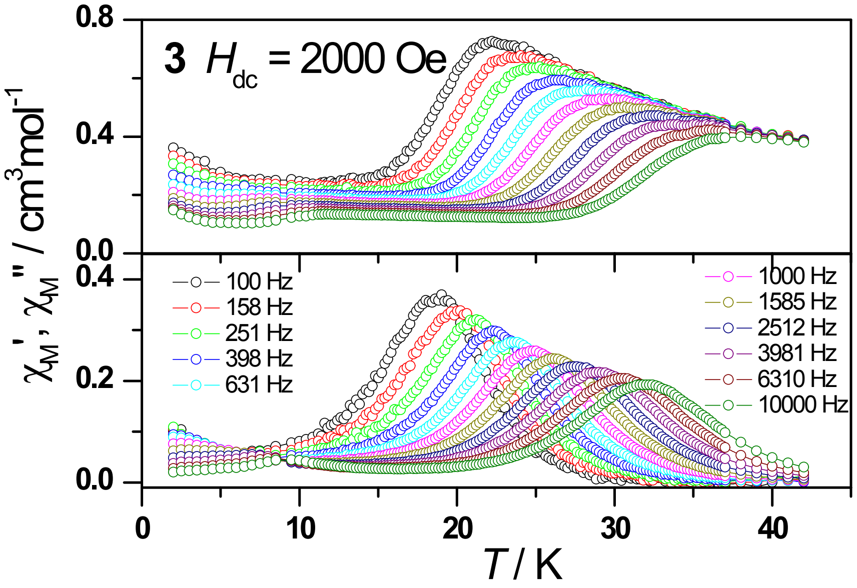

(b)

Fig. S9 The field dependence and temperature dependence of ac susceptibility at 2K for 1 kHz (a) and under 2 kOe field (b) for **3.**

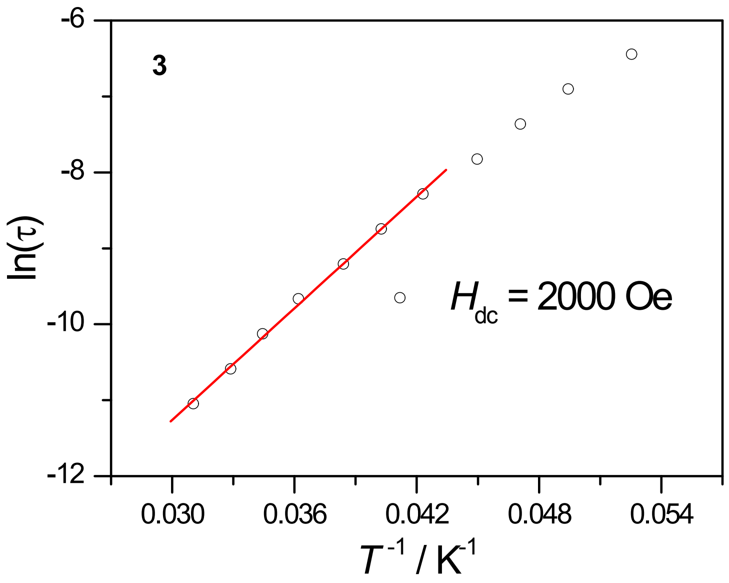

Fig. S10 Plots of lnτ vs. T^{-1} for **3** under 2 kOe dc field. The red lines show the fitting results according to the Arrhenius law.

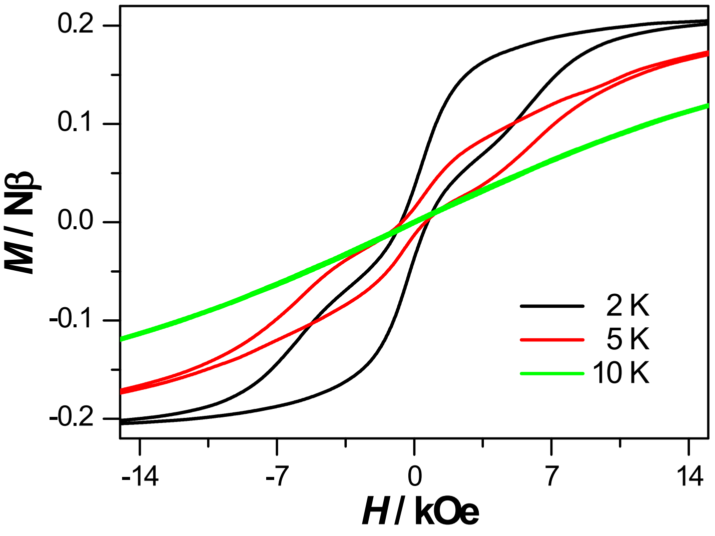

a)

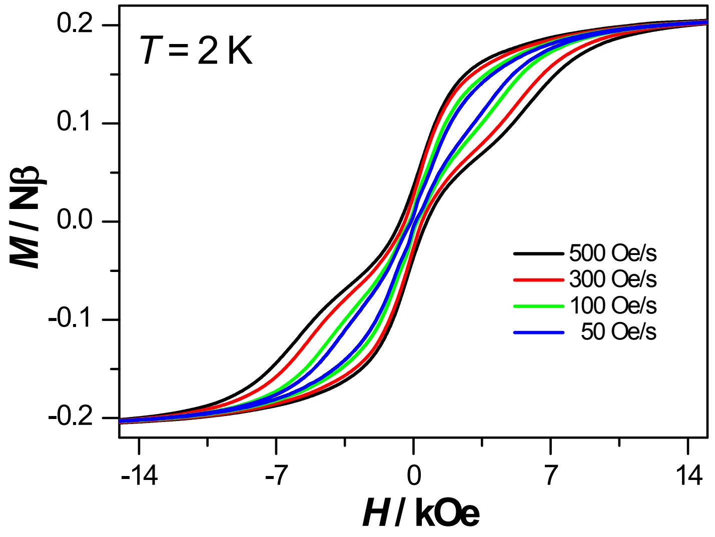

b)

Fig. S11 Hysteresis loop for **3** measured at different temperatures with sweep rates of 500 Oe/s (a) and different sweeping rates at 2 K (b).

Computational details

From Fig. 1, there is one type of Dy^{3+} ion, and thus we only need to calculate one Dy^{3+} fragment. Complete-active-space self-consistent field (CASSCF) calculations on individual lanthanide Dy^{3+} fragment of the model structure (inset of Fig.2) extracted from complex **1** on the basis of X-ray determined geometry have been carried out with MOLCAS 7.8 program package.[S1]

During the calculations, the other Dy^{3+} ions for each complex were replaced by diamagnetic Lu^{3+}. The basis sets for all atoms are atomic natural orbitals from the MOLCAS ANO-RCC library: ANO-RCC-VTZP for Dy^{3+} ions; VTZ for close O; VDZ for distant atoms. The calculations employed the second order Douglas-Kroll-Hess Hamiltonian, where scalar relativistic contractions were taken into account in the basis set and the spin-orbit couplings were handled separately in the restricted active space state interaction (RASSI-SO) procedure. For the fragment of Dy^{3+}, active electrons in 7 active spaces include all *f* electrons (CAS(9 in 7) in the CASSCF calculation. We have mixed the maximum number of spin-free state which was possible with our hardware (all from 21 sextets, 128 from 224 quadruplets, 130 from 490 doublets for the Dy^{3+} fragment).

To fit the exchange interactions in four complexes, we took two steps to obtain them. Firstly, we calculated one Dy^{3+} fragment using CASSCF to obtain the corresponding magnetic properties (see the first part). And then, the exchange interaction between the magnetic centers is considered within the Lines model,[S2] while the account of the dipole-dipole magnetic coupling is treated exactly. The Lines model is effective and has been successfully used widely in the research field of f-element single-molecule magnets.[S3]

For complex **1**, we only consider three types of J (see Figure S12) and the intermolecular interaction zJ' was set to 0.0 cm^{-1}.

The exchange Hamiltonian is:

$$\hat{H}_{exch} = -J_1^{total}(\hat{S}_{Dy1}\hat{S}_{Dy2} + \hat{S}_{Dy1}\hat{S}_{Dy3}) - J_2^{total}\hat{S}_{Dy1}\hat{S}_{Dy4} - J_3^{total}\hat{S}_{Dy3}\hat{S}_{Dy4} \quad (S1)$$

The J_1^{total}, J_2^{total} and J_3^{total} are the parameters of the total magnetic interaction (

$J^{total} = J^{diploar} + J^{exchange}$) between magnetic center ions. The $\hat{S}_{Dy}$ = ±1/2 are the ground pseudo-spin on the Dy^{3+} sites. The dipolar magnetic coupling can be calculated exactly, while the exchange coupling constants were fitted through comparison of the computed and measured magnetic susceptibility and molar magnetization using the POLY_ANISO program.[S4]

Table S5. Energies (cm^{-1}) and ***g*** (g_x, g_y, g_z) tensors of the lowest spin-orbit states on one Dy^{3+} fragment of **1**.

Energy of eight lowest KDs	*g* tensor of the ground KD
0.0	g_x = 0.0018
189.9	g_y = 0.0024
377.3	g_z = 19.8294
479.1	
557.4	
641.8	
716.2	
776.3	

Table S6. The calculated charges of coordination oxygen atoms around Dy^{3+}.

	O1	O2	O3	O4	O4	O5	O5	O6
charge	-0.8126	-0.7865	-0.9226	-1.0380	-0.6635	-0.7927	-1.0654	-0.9268

Table S7. Exchange energies (cm^{-1}) and main values of the g_z for the 8 lowest exchange doublets.

	Energy	g_z
1	0.000	39.658
2	0.960	0.027
3	0.974	79.317
4	1.934	39.658
5	2.829	0.033
6	3.717	39.658
7	3.788	39.658
8	4.676	0.001

Table S8. Parameters of the magnetic interactions between Dy^{3+} ions in **1** (cm^{-1})

	J_1	J_2	J_3
$J^{dipolar}$	4.2	0.67	0.64
J^{exch}	2.0	-1.25	-0.25
J	6.2	-0.58	0.39

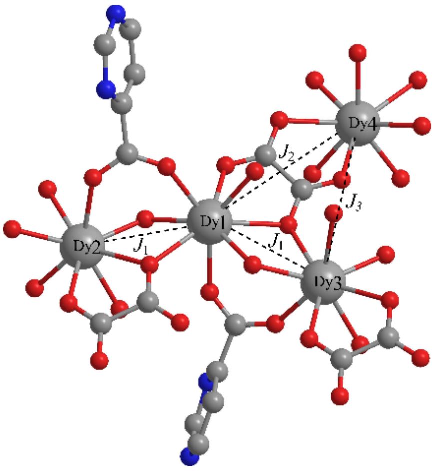

Fig. S12. Three types of J_1, $J_2$and J_3in complex **1**.

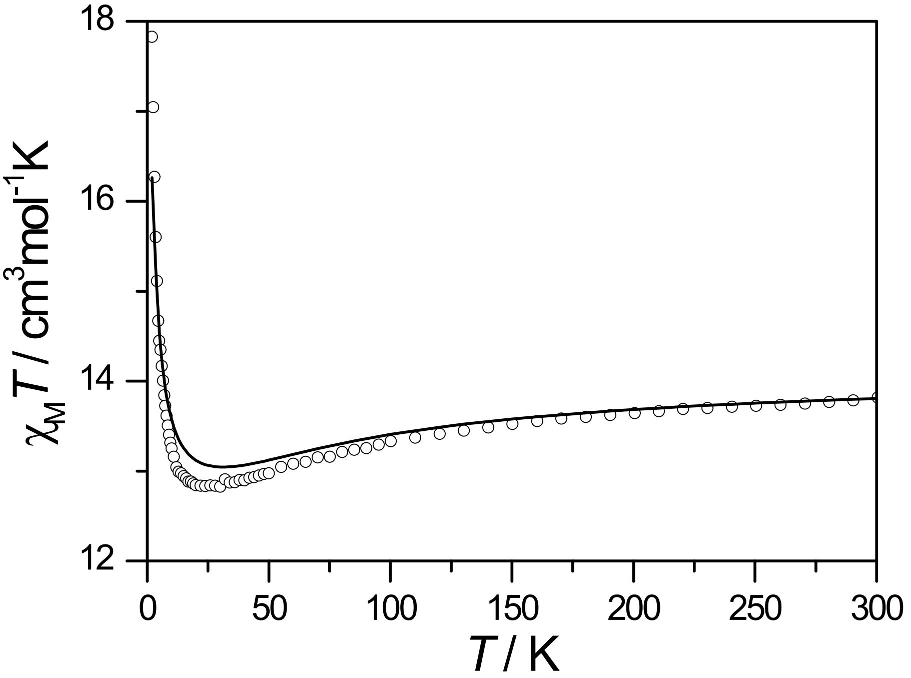

Fig. S13 A comparison of the experimental and calculated (solid line) magnetic susceptibility of complex **1**.

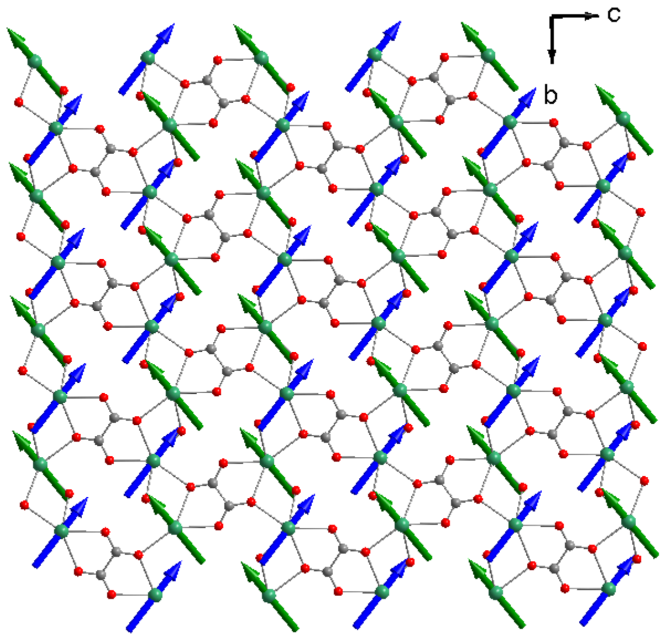

Fig. S14 The orientation of easy axis of **1** within the 2D layer.

References:

S1 G. Karlström, R. Lindh, P. Å. Malmqvist, B. O. Roos, U. Ryde, V. Veryazov, P. O. Widmark, M. Cossi, B.

Schimmelpfennig; P. Neogrady, L. Seijo, MOLCAS: a Program Package for Computational Chemistry. *Comput. Mater. Sci*. 2003, **28**, 222.

S2 M. E. Lines, *J. Chem. Phys*. 1971, **55**, 2977.

S3 (a) K. C. Mondal, A. Sundt, Y. H. Lan, G. E. Kostakis, O. Waldmann, L. Ungur, L. F. Chibotaru, C. E. Anson, A. K. Powell, *Angew. Chem. Int. Ed*. 2012, **51**, 7550. (b) S. K. Langley, D. P. Wielechowski, V. Vieru, N. F. Chilton, B. Moubaraki, B. F. Abrahams, L. F. Chibotaru, K. S. Murray, *Angew. Chem. Int. Ed*. 2013, **52**, 12014.

S4 (a) L. F. Chibotaru, L. Ungur, A. Soncini, *Angew. Chem. Int. Ed.*, 2008, **47**, 4126. (b) L. Ungur, W. Van denHeuvel, L. F. Chibotaru, *New J. Chem.*, 2009, **33**, 1224. (c) L. F. Chibotaru, L. Ungur, C. Aronica, H. Elmoll, G. Pilet, D. Luneau, *J. Am. Chem. Soc.*, 2008, **130**, 12445.
